# Polycystic Ovary Syndrome and Labor Market Attachment: Sequence Analysis

**DOI:** 10.3389/ijph.2025.1607889

**Published:** 2025-04-14

**Authors:** Beata Vivien Boldis, Ilona Grünberger, Jonas Helgertz, Agneta Cederström

**Affiliations:** ^1^ Department of Public Health Sciences, Stockholm University, Stockholm, Sweden; ^2^ Epidemiology, Population Studies and Infrastructures (EPI@LUND), Lund University, Lund, Sweden; ^3^ Centre for Economic Demography, Lund University School of Economics and Management, Lund, Sweden; ^4^ Department of Economic History, Lund University, Lund, Sweden

**Keywords:** PCOS, labor market attachment, sequence analysis, sickness benefit, unemployment

## Abstract

**Objectives:**

Polycystic ovary syndrome (PCOS) is an endocrine disorder in women of fertile age which may also affect the labor market attachment. We investigated labor market attachment trajectories among working age women diagnosed with PCOS.

**Methods:**

A cohort of 157,356 women born in 1975–1977 were followed annually between the ages of 30 and 39, using data from Swedish administrative registers. Multinomial logistic regression was employed to assess associations between being diagnosed with PCOS (after the age of 15) and belonging to the identified clusters of labor market attachment trajectories.

**Results:**

Women with PCOS spent less time in employment and were more dependent on sickness benefits during the follow-up time than those without PCOS. Five labor market attachment clusters were identified: *stable employment, education into employment, labor market exclusion, continuously unstable position, long-term sickness*. Compared to being in the *stable employment* cluster, women diagnosed with PCOS were more likely to experience *long-term sickness* [RRR (relative risk ratio): 1.97 (CI: 1.90–2.05)], and *education into employment* [RRR: 1.11 (CI: 1.07–1.15)].

**Conclusion:**

PCOS can lead to disadvantaged labor market outcomes. Better strategies are needed to prevent economic exclusion among women diagnosed with PCOS.

## Introduction

The socioeconomic consequences of women’s reproductive health at various stages, including pregnancy, menstrual health, and menopause, have been extensively studied [1, 2]. A prominent example is derived from the 1958 British Cohort study, which demonstrated a correlation between severe menopausal symptoms and decreased employment rates [1]. Furthermore, recent systematic reviews and meta-analyses have highlighted the profound negative impact of menstrual disturbances, such as polycystic ovary syndrome (PCOS), on academic performance and career progression [3, 4].

PCOS is a common endocrine disorder that affects 5%–20% of women in their reproductive ages [5–7]. It is characterized by ovulatory dysfunction, hyperandrogenism, and visible polycystic ovaries on ultrasound in the absence of any mimicking conditions such as Cushing’s syndrome, Turner’s syndrome, androgen secreting tumors or congenital adrenal hyperplasia [8]. Research has demonstrated that PCOS is associated with several reproductive, cardiovascular, metabolic, psychological and dermatological conditions [9–13], and impaired overall quality of life [14]. Having PCOS during pregnancy is associated with negative neonatal outcomes and pregnancy complications [15–17]. PCOS is a chronic illness which underscores the necessity for longitudinal management strategies including mitigating the potential long-term health consequences and fertility concerns. In addition, there is potential heterogeneity in how PCOS clusters with comorbidities across socioeconomic groups.

Despite the relatively high prevalence of PCOS and its many comorbidity conditions, the etiology is still poorly understood. Results from previous research suggests that PCOS possibly is the result of combination of genetic susceptibility along with certain environmental and social exposures [18–20]. While PCOS is traditionally investigated as a purely metabolic condition, a growing body of evidence highlights the importance of studying PCOS from a social perspective. A previous study in the US has found evidence for an association between low childhood socioeconomic position (SEP) and PCOS, especially among women with higher education [21]. From the Nordic context, a recent Danish register-based cohort study found that incidence of PCOS is highest among women with low SEP [22]. Our previous study in Sweden [23] found evidence that women of foreign-born mothers had a higher risk for being diagnosed with PCOS.

Two recent studies have shed light on the serious negative impact of menstrual disturbances, including PCOS, on individual’s academic life and career advancement [3, 4]. Many women of reproductive age experience a decline in academic performance and in their quality of life due to menstrual disturbances [3]. A Finnish cohort study found an association between PCOS and lower participation in working life and for a higher risk of disability retirement among PCOS women [24]. This body of evidence suggests that burdensome conditions such as PCOS which only affects the female population can contribute to a widening gender gap in health.

While most of the research has focused on how low SEP contributes to the development of PCOS, there is increasing concerns on the extent to which women who are diagnosed with PCOS experience different career trajectories during their peak working ages. A recent editorial from The Lancet Regional Health Europe, 2002 [25], highlights the economic burden of PCOS, both at an individual level and for the healthcare system. To our knowledge, this study is the first to examine the career trajectories of women diagnosed with PCOS compared to women without the diagnosis. Furthermore, we also control for other labor market influencing factors such as attained education, county of origin, civil status and number of children. The overarching aim of the current study is to investigate how being diagnosed with PCOS changes the career trajectories of women.

## Methods

### Study Population

**FIGURE 1:**
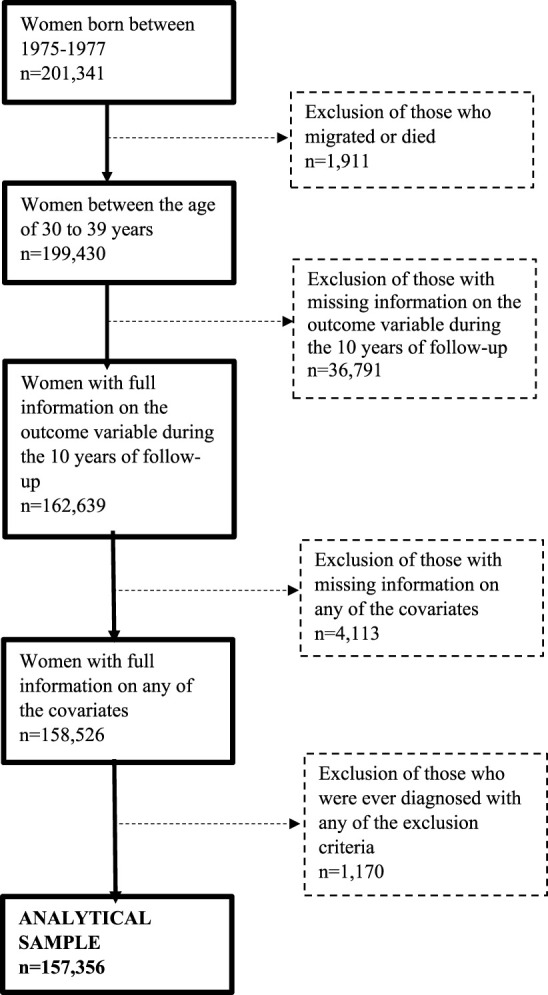
Flow diagram of the study sample creation (Sweden, 2025).

This study used data from the Swedish Interdisciplinary Panel (SIP-ENTRY), administered at the Centre for Economic Demography at Lund University. The baseline study population consisted of all women born between 1975–1977 who continuously reside in Sweden between the ages 30 and 39 (n = 201,341). Consequently, women who migrated or died during this interval were excluded (n = 1,911). Additionally, we excluded women with missing data on the outcome variable during any of the follow-up years (n = 36,791), or if they had missing information on any of the covariates (see *section Covariates*) used in the analysis (n = 4,113). Lastly, women who ever were diagnosed with any of the mimicking diseases that serve as exclusion criteria (see section *Exclusion criteria*) (n = 1,170) were omitted from the sample. This resulted in a final study population of 157,356 women, illustrated in Figure 1.

### Outcome: Labor Market Attachment

Woman’s labor market attachment was determined by using different sources of income available in the Income and Taxation Register (IoT) (Supplementary Table SA1), between 2004 and 2016. Through careful harmonization of the income variables, we identified incomes obtained through six different activities; i) labor income, ii) unemployment and/or welfare income, iii) childbirth during the current year, iv) student benefits and loans, v) sickness and disability benefits, and vi) other sources of income.

An initial condition for being classified as in *employment* during a given year was if the labor income exceeded three price base amounts, provided by Statistics Sweden (SCB) [26]. This threshold was selected to ascertain that the individual’s activity in the labor market exceeded a certain minimum level. Secondly, the individual could not have given birth to a child during the year, nor received sickness/disability benefits, student benefits/loans or unemployment benefits that amounted to 25% of their labor income.

*Parental status* was defined as giving birth to a child in the given year. *Unemployment status* was defined as the individual not being in employment nor parental status and receiving positive unemployment benefits that exceed those obtained from sickness/disability or student benefits and loans. The sickness and student statuses were generated in an analogous fashion, however naturally dependent on the income received from the relevant source exceeding the other two. Lastly, the *other* status was defined as not being in either of the aforementioned categories, yet being recorded in the income register.

### Exposure: PCOS

PCOS was identified through the Swedish National Patient Registry (NPR) [27] after the age of 15, from both inpatient and outpatient diagnoses (E28), with the vast majority of the diagnoses coming from outpatient data (99.7%) [23]. Until 2012, the aggregated ICD-10 code E28 was used, due to no access to the more detailed PCOS diagnosis code (ICD-10: E28.2). The PCOS code E28.2 constitutes the vast majority – 82%, of diagnoses within the E28 category in the data for the period of 2012–2016. After 2012, the more detailed 4-digit E28.2 ICD-10 code was also available to identify PCOS cases. For women who were diagnosed with the more aggregated code before 2012, and then with the detailed E28.2 during the later years of follow-up, their later diagnosis code was used. Considering that PCOS is a chronic illness, a diagnosis of PCOS recorded anytime was used as a proxy for always having had the condition.

#### Exclusion Criteria

Women diagnosed with conditions that could cause similar symptoms as PCOS, including Turner syndrome (Q96), Malignant neoplasm of ovary (C56), Suprarenal tumor (C74), Adrenogenital syndrome (E25), Cushing disease (E24) and Pituitary hypersecretion (E22), have been excluded to ensure specificity, in accordance with our previous research [23]. Both main and contributing diagnoses were considered. Thus, the study population only included women who were never diagnosed with any of the exclusion criteria.

### Covariates

Information on civil status and region of origin were obtained from the Total Population register (TPR). *Civil status* was coded into married/in a registered relationship and into not married/not in a registered relationship. *Region of origin* was categorized as a dichotomous variable, distinguishing between those born in and outside of Sweden. *Highest attained education* was obtained from the Register of Participation in Education (UREG) and grouped into three categories: primary, secondary, and post-secondary. *Number of children* were calculated by retrieving information from TPR on the total number of children born to the same woman, and were grouped into no children or, one or more children. *Maternal and paternal region of origin* was obtained from the TPR and grouped as a three-level variable: Sweden, Outside of Sweden, and Missing. *Maternal and paternal highest attained education* were retrieved from UREG, where we distinguish between: Post-secondary, Primary/Secondary and Missing. Information on all covariates were measured at the age of 29 years and used as time-invariant variables, to minimize temporal variability, in the analysis.

### Statistical Analysis

In an initial descriptive analysis, Pearson’s chi-square test and t-tests were used to assess whether differences between the populations of women with and without PCOS diagnoses were statistically significant.

We proceeded to use sequence analysis [28, 29] to identify clusters of labor market attachment trajectories. This was based on the 10-year outcomes observed for each individual in the sample, using the definitions of labor market position defined earlier. We used an optimal matching (OM) algorithm with an INDELSLOG cost matrix to compute distances between sequences, a choice of algorithm which flexibly and systematically aligns sequences by using data-driven transition costs that emphasize the timing, ordering, and duration of states, ensuring sensitivity to significant transitions [30]. The sequences were then clustered using an agglomerative hierarchical clustering algorithm with Ward’s minimum variance method. Using different diagnostic tools such as average silhouette width (ASW) an optimal number of clusters were identified. Each woman was then assigned a cluster membership to which her individual sequence belonged.

Whether a woman was diagnosed with PCOS was used as the main exposure while cluster membership was used as the outcome variable in multinomial logistic regression models. Model 1 estimated the unadjusted association between PCOS diagnosis and cluster membership, whereas Model 2 included the covariates civil status, highest attained education, region of origin and number of children, all measured at the age of 29. Data management was done with STATA/MP 17.0 (StataCorp) while data analyses were carried out in R 4.4.0. with help of the TraMineR library [31].

## Results

A total of 157,356 women were included in this study, of whom 2% (n = 3,077) were diagnosed with PCOS anytime during the follow-up period. Among these women, 79% were born in Sweden, 32% were married or cohabitating, 54% had post-secondary education and 35% had at least one or more children. Table 1 summarizes the share of observation time in the different labor market states and other descriptive characteristics of the study population. Women with PCOS diagnoses spent slightly less time in employment (57.6%), compared to those without PCOS diagnoses (61.8%). Also, women with PCOS spent more time on sickness benefits (11.5%), than those without PCOS (6.9%).

**TABLE 1:** Descriptive characteristics of the study population, stratified by women with and without polycystic ovary syndrome diagnosis, n = 157,356 (Sweden, 2025).

	With PCOS diagnoses n = 3,077	Without PCOS diagnoses n = 154,279	
Dependent variable: Share of observation time (age 30–39 years) in labor states			p-value^a^
Parental leave	8.9%	9.5%	<0.01
Employed	57.6%	61.8%	<0.01
Unemployed	6.4%	6.0%	<0.01
Sickness	11.5%	6.9%	<0.01
Studying	5.8%	5.0%	<0.01
Other	9.8%	10.8%	<0.01
Independent variables: Observed at the age of 29 years			p-value^a^
*Civil status*			
Married	32.3%	28.1%	<0.01
Not married	67.7%	71.9%	
*Highest attained education*			
Primary	8.0%	6.3%	<0.01
Secondary	38.0%	38.8%	
Post-secondary	54.0%	54.9%	
*Region of origin*			
Sweden	78.8%	83.2%	<0.01
Outside of Sweden	21.2%	16.8%	
*Number of children*			
No children	65.0%	51.2%	<0.01
One or more children	35.0%	48.8%	
*Maternal region of origin*			
Sweden	82.9%	86.2%	<0.01
Outside of Sweden	12.1%	10.8%	
Missing	5.0%	3.0%	
*Paternal region of origin*			
Sweden	82.9%	86.4%	<0.01
Outside of Sweden	12.0%	10.6%	
Missing	5.1%	3.0%	
*Maternal highest attained education*			
Primary/Secondary	59.6%	61.7%	<0.01
Post-secondary	24.4%	25.3%	
Missing	16.0%	13.0%	
*Paternal highest attained education*			
Primary/Secondary	61.6%	62.8%	<0.01
Post-secondary	19.6%	21.2%	
Missing	18.8%	16.0%	

^a^ Denotes a p-value between *With PCOS, diagnosis* and *Without PCOS, diagnosis*.

**FIGURE 2:**
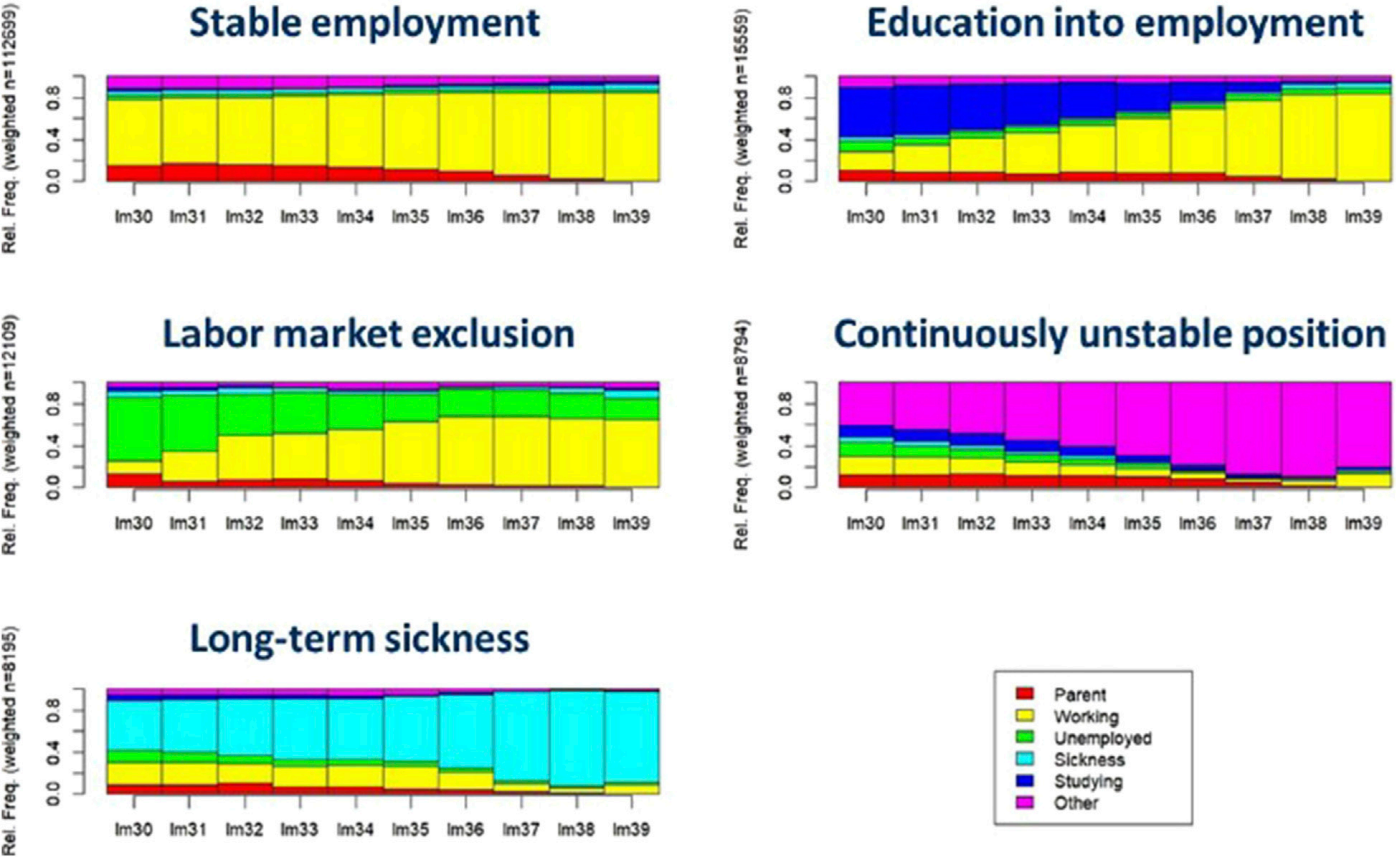
Density plots of sequences of labor market positions among Swedish women born between 1975–1977 and followed from age 30 to 39 (Sweden, 2025). Note: i. Lm denotes labor market position at a given age. For example, “lm30” send for Labor market position at age 30.

Labor market trajectories were best captured by five clusters and categorized as: *stable employment* (T1), *education into employment* (T2), *labor market exclusion* (T3), *continuously unstable position* (T4), *long-term sickness* (T5). Figure 2 illustrates the state frequencies of these five clusters over the age scale. The quality of the partitions for different numbers of clusters for the different indicators is shown graphically in Supplementary Figure SA1.

The most common trajectory was stable employment (T1), containing 112,699 women (71.6%) and also used as the reference category in the multivariate analysis. It is characterized by a short period of parental leave and stable employment throughout most of the period. The second most common trajectory was education into employment (T2) with 15,599 women (9.9%). This trajectory differed from the first trajectory by having a longer period of education in the early 30s, then transitioning to stable employment. The labor market exclusion (T3) was the third largest cluster of trajectories (n = 12,109, 7.7%). It is characterized by the highest level of unemployment that slowly goes over to employment although not completely. The least common trajectories (T4, T5), which contain fairly similar number of individuals (n = 8,974 (5.7%), n = 8,195 (5.2%) respectively), were continuously unstable position and long-term sickness. Women in these clusters had severe labor market difficulties either with non-traceable income or relied on sickness benefits. Supplementary Table SA2 summarizes cluster membership for women with and without PCOS diagnosis and for explanatory variables.

Results from the multinomial logistic regression models are shown in Table 2 with estimates of the association between being diagnosed with PCOS and the clusters of labor market trajectories. Results from the unadjusted associations showed that women with PCOS have a 97% higher risk [relative risk ratio (RRR): 1.97 (CI: 1.90–2.05)] of experiencing long-term sickness across the labor market (T5), an 11% higher risk [RRR: 1.11 (CI: 1.07–1.15)] of remaining in a longer period of education (T2), and a 4% higher risk [RRR: 1.04 (CI: 1.00–1.09)] of experiencing labor market exclusion (T3) compared to those with trajectories in the stable employment cluster.

**TABLE 2:** Unadjusted and adjusted results from multinomial logistic regression models (Cluster 1 as reference: stable employment), (Sweden, 2025).

	Cluster 2 (ref 1) *education into employment*		Cluster 3 (ref 1) *labor market exclusion*		Cluster 4 (ref 1) *continuously unstable position*		Cluster 5 (ref 1) *long-term sickness*	
	UA^a^	A^b^	UA^a^	A^b^	UA^a^	A^b^	UA^a^	A^b^
PCOS (REF: PCOS)								
Ever PCOS	1.11 [1.07–1.15]	1.18 [1.13–1.22]	1.04 [1.00–1.09]	1.11 [1.05–1.16]	0.96 [0.91–1.01]	0.73 [0.68–0.78]	1.97 [1.90–2.05]	1.97 [1.89–2.06]
Civil status (REF: Married)								
Not married		1.39 [1.37–1.41]		1.43 [1.40–1.45]		0.90 [0.88–0.92]		1.13 [1.11–1.15]
Highest attained education (REF: Post-secondary)								
Primary		0.18 [0.17–0.19]		5.08 [4.96–5.22]		4.88 [4.73–5.04]		11.3 [11.0–11.6]
Secondary		0.20 [0.19–0.21]		2.17 [2.13–2.20]		1.72 [1.69–1.76]		2.83 [2.78–2.88]
Region of origin (REF: Sweden)								
Outside of Sweden		0.95 [0.91–0.99]		1.41 [1.35–1.46]		1.18 [1.13–1.23]		1.05 [1.00–1.10]
Children (REF: One or more children)								
No children		0.47 [0.46–0.48]		0.64 [0.63–0.65]		1.93 [1.89–1.97]		1.11 [1.09–1.13]
Maternal region of origin (REF: Sweden)								
Outside of Sweden		1.20 [1.17–1.23]		1.15 [1.12–1.18]		1.55 [1.50–1.59]		1.09 [1.06–1.13]
Missing		1.30 [1.21–1.38]		1.09 [1.01–1.17]		1.90 [1.76–2.09]		0.88 [0.81–0.96]
Paternal region of origin (REF: Sweden)								
Outside of Sweden		1.12 [1.09–1.14]		1.22 [1.19–1.25]		1.17 [1.13–1.21]		1.15 [1.12–1.18]
Missing		1.51 [1.43–1.60]		1.37 [1.28–1.46]		1.52 [1.42–1.63]		1.28 [1.18–1.38]
Maternal highest attained education (REF: Post-secondary)								
Primary, Secondary		1.27 [1.25–1.29]		1.20 [1.17–1.22]		0.78 [0.76–0.79]		1.04 [1.02–1.06]
Missing		1.64 [1.55–1.73]		1.40 [1.32–1.48]		0.99 [0.93–1.06]		1.24 [1.17–1.32]
Paternal highest attained education (REF: Post-secondary)								
Primary, Secondary		1.29 [1.27–1.31]		1.19 [1.16–1.21]		0.78 [0.76–0.80]		1.10 [1.08–1.12]
Missing		1.65 [1.60–1.71]		1.43 [1.38–1.49]		0.98 [0.93–1.02]		1.33 [1.28–1.39]

^a^ UA, Unadjusted.

^b^ A, Adjusted.

The fully adjusted model indicated that women with only primary education (RRR: 5.08, [CI: 4.96–5.22]) were at highest risk of experiencing labor market exclusion or long-term sickness [RRR: 11.3 (11.0–11.6)], compared to those in stable employment. Additionally, women who were born outside of Sweden were more likely to end up with labor market exclusion [RRR: 1.41 (CI:1.35–1.46)]. Women without children were at higher risk for being in continuous unstable position [RRR: 1.93, (CI:1.89–1.97)] and in long-term sickness [RRR: 1.11, (1.09–1.13)], compared to women with children. Women with a mother born outside of Sweden were at the highest risk to remain in continuously unstable position [1.55 (1.50–1.59)], compared to those with a mother born in Sweden. Women with a father born outside of Sweden were at highest risk to remain in labor market exclusion [1.22 (1.19–1.25)], compared to those with a father born in Sweden. Women with a mother or father who only had primary or secondary education were at higher risk for remaining in education longer [1.27 (1.25–1.29) and 1.29 (1.27–1.31)], compared to those with a mother or father with post-secondary education.

The observed unadjusted associations between PCOS and being in the education into employment cluster or labor market exclusion cluster became a slightly bit stronger [RRR:1.18 (1.13–1.22) and 1.11 (1.05–1.16), respectively] after adjusting for civil status, education, region of origin, number of children, maternal and paternal education and region of origin. Controlling for these variables partially attenuated the risk for PCOS as the adjusted model results showed a slightly lower risk for being in continuous unstable position [RRR: 0.73, (CI:0.68–0.78)]. However, these control variables did not affect the observed risk of being in the long-term sickness [RRR: 1.97, (CI: 1.89–2.06)] clusters.

## Discussion

This cohort study investigated labor market trajectories among women diagnosed with PCOS during early adulthood. The findings support our underlying hypothesis that PCOS can negatively influence the labor market outcomes of early mid-life women, while we also confirmed higher risks of disadvantage, in terms of carrier patterns, among those with only primary education attained or those with immigrant background.

Similarly to a Danish study [22] which found evidence that women with PCOS had more often experienced unemployment and reliance on welfare, and that immigrants with PCOS had a higher risk for low SEP [22]. This indicates that women with PCOS diagnoses tend to experience labor market difficulties while women without the condition have an advantage on establishing a stable labor market position.

Interestingly, an American study by Merkin et al [21] found that women with a high education had an increased risk for PCOS development, especially those brought up in a low socioeconomic environment, compared to women with lower education. The authors argued that PCOS women with high individual SEP but low parental SEP had a higher chance of experiencing adversity during childhood and peripubertal age affecting their hormonal and menstrual cycles [21]. It is plausible that women with a higher SEP might be more likely to seek healthcare earlier or to a greater extent, and thus also receive a PCOS diagnosis for milder forms of the disease. Furthermore, if the condition is more often treated or better managed among women with higher education, this could also explain their resilience against labor marker adversity. It is thus not necessarily that the prevalence of PCOS is higher among women with high SEP but more likely that women with low SEP can go undiagnosed longer. Higher educational attainment promotes health-seeking behavior and compliance with medical advice, which may also lead to a more successful PCOS treatment. Moreover, based on our results women with PCOS and higher attained education are more resilient against labor market adversity.

The current results need to be interpreted within the context of multimorbidity associated with PCOS. As reported in our previous study [32] based on the same cohorts as this given study, PCOS is a risk factor for multiple comorbidity conditions such as obesity, depression, anxiety, and eating, sleeping and/or sexual disorders. Similarly, several other studies have reported comorbidity conditions in PCOS including a myriad of reproductive, cardiovascular, metabolic, psychological and dermatological conditions [9–13, 33]. Findings from a recent Australian study [34] suggest that the onset of comorbidity conditions may start earlier in the life trajectory for women with PCOS compared to those without, thereby leading to prolonged health risks. It is also important to acknowledge, that the clustering of multimorbidity, such as musculoskeletal disorders, diabetes, cardiovascular disease or asthma, has been previously associated with negative impact on work and unemployment [35], which resonate with our results. The prevalence of PCOS is on its rise and causes an economic burden for both individuals and for the society [25].

Individuals with lower SEP often experience greater exposure to stressors, including limited access to healthcare and restricted material resources. These challenges can increase the risk of developing chronic conditions, potentially including PCOS. As such, lower SEP can serve as a precursor to poor health outcomes [36].

However, as the result of this present study showed. PCOS can also limit individuals’ social and economic opportunities. For instance, menstrual irregularities and other PCOS-related challenges might hinder the ability to achieve educational goals [3] or fully engage in the workface [22]. PCOS has also been associated with lower earnings and a greater likelihood of disability-related retirement [24]. Thus, PCOS not only has the potential to result from lower SEP but may also exacerbate socioeconomic disadvantage, creating a cycle of inequality.

### Strengths and Limitations

A major strength of this study is the use of extensive, nationally representative Swedish register data, enabling the tracking of labor market trajectories from 2004 to 2016 for women born between 1975 and 1977. This high-quality registry data covers a broad sample of women, providing comprehensive sociodemographic, socioeconomic, and health-related information on the entire Swedish population [27, 37].

The estimated prevalence of PCOS clearly depends on the source of data and diagnostic criteria used and it ranges somewhere between 8% and 18% [7]. We also know from our previous studies [23, 32] that the prevalence of PCOS in the Swedish NPR is lower than it would be expected based on population surveys or clinical studies. One important reason for the low diagnoses rate could be the lack of clear diagnostic criteria.

A limitation of this study is that we only had access to ICD codes at the three-digit level of aggregation in both inpatient and outpatient data prior to 2012. As a result, we were only able to study ovarian dysfunction (E28) rather than PCOS (E28.2), which is the most common subcategory under E28. However, we do not believe this poses a significant issue, as PCOS constitutes the vast majority (82%) of diagnoses within the E28 category. For women initially diagnosed with the more aggregated E28 code before 2012 and later with the more specific E28.2 during follow-up, we used their updated diagnosis. Given that PCOS is a chronic condition, any recorded diagnosis of PCOS at any point in time was considered indicative of having the condition throughout.

Since the mean age of first PCOS diagnoses in Sweden is 26 years [23], our follow-up window allows for most women who would develop the condition to be included in our study by starting the follow-up at the age of 30 years. Secondly, the age-spectrum of 30–39 years represents early mid-life, which is often characterized by relatively stable labor market position and childbearing in the general population. This implies that trajectories involving transitions between employment and parental leave for women should not be categorized as unfavorable. We focused on a cohort of women observed during a relatively short time period, in terms of calendar years which gave the advantage of analyzing a more homogenous group of women. The risk of bias due to macroeconomic fluctuations that arise from changes in the broader economic environment, is low in our study compared to studies since we cover a 10-year period. One could question the generalizability of the findings to younger age cohorts since the population composition has changed with the increased immigration to Sweden in the last decade. However, even if the population composition is changing, we can still gain a deeper understanding of how PCOS can negatively influence labor market outcomes of early mid-life women from our results.

Additionally, the observed time-varying explanatory variables were measured at the age of 29 years and treated as time-fixed throughout the analysis. Consequently, we may not have captured additional changes in these explanatory variables occurring beyond the age of 29. However, this approach reduces bias in the relationship between the observed explanatory variables and labor market trajectories, ensuring a more consistent interpretation of their influence.

Another limitation of this study is the potential for residual confounding due to the limited availability of explanatory variables. While we included a range of explanatory variables to account for possible confounders, it is likely that unmeasured or unknown factors could influence our findings. In such case, the observed associations may partly reflect the effect of unmeasured confounders.

Sequence analysis has been previously used in a longitudinal life-course study, utilizing yearly income data [38, 39]. One potential limitation lies in the methodology used to construct and categorize clusters of trajectories into five distinct groups, each based on the individuals’ primary source of income. These five clusters were determined using diagnostic criteria for cluster membership. The main limitation, however, lies in the somewhat arbitrary selection of these five specific clusters, rather than in the total number of clusters chosen.

This article is an original contribution to the understanding of social and economic aspects of PCOS. Although there is previous research which explores the relationship between SEP and PCOS, there is also need for research which addresses the dynamic relations between PCOS and adverse economic and labor market outcome viewed across the life-course. PCOS affects women from all social strata and backgrounds, however, the individual ability how women can cope with their symptoms and comorbidities may differ. A recent German prospective panel study found that individuals with obesity have difficulties to reenter the labor market after a period of unemployment and that being unemployed increases their risk of obesity [40–42]. Considering that visceral adiposity amplifies the reproductive and metabolic outcomes of PCOS [43], we might need to target the negative outcomes of PCOS from different angles, also by helping PCOS women reentering the labor market, consequently lowering the risk of further comorbidity amplification. Further research is therefore needed to explore the underlying mechanism behind childhood SEP’ impact on adult life PCOS and comorbidity conditions, and how these negative health outcomes are related to early mid-life labor market difficulties.

### Conclusion

This study investigated the consequences of having been diagnosed with PCOS on women’s working career and labor market activity. Although most women in our cohort experienced a labor market entry with stable employment, women with PCOS were more likely to experience adverse labor market outcomes such as labor market exclusion or long-term sickness. Since PCOS can lead to labor market disadvantage, increased awareness and early detection of PCOS can help equalize economic conditions for patients.

## Data Availability

Swedish law prohibits the distribution of and unauthorized access to the data used for this study, stored and analyzed on a secure server managed by Statistics Sweden (https://www.scb.se/en/services/ordering-data-and-statistics/ordering-microdata/mona--statistics-swedens-platform-for-access-to-microdata/about-mona/).
