# Supplementary material for: Polycystic Ovary Syndrome and Labor Market Attachment: Sequence Analysis
Source: Int J Public Health. 2025 Apr 14;70:1607889. doi: 10.3389/ijph.2025.1607889 (PMC12034860; doi:10.3389/ijph.2025.1607889)
Supplement: Supplementary file 1 [file DataSheet1.docx]

APPENDICES

Appendix Table 1. Variables used from the Income and Taxation Register (**Sweden, 2025**).

|  | **2004** | **2005** | **2006** | **2007** | **2008** | **2009** | **2010** | **2011** | **2012** | **2013** | **2014** | **2015** | **2016** |  |
| --- | --- | --- | --- | --- | --- | --- | --- | --- | --- | --- | --- | --- | --- | --- |
| **Employed** | | | | | | | | | | | | | | |
| CARB - Arbetsinkomst | **X** | **X** | **X** | **X** | **X** | **X** | **X** | **X** | **X** | **X** | **X** | **X** | **X** |  |
| CBEFVI - Beskattningbar försvarsinkomst | **X** | **X** | **X** | **X** | **X** | **X** | **X** | **X** | **X** | **X** | **X** | **X** | **X** |  |
| CSFVI - Sammanräknad försvärsinkomst* | **X** | **X** | **X** | **X** | **X** | **X** | **X** | **X** | **X** | **X** | **X** | **X** | **X** |  |
| TKULON - Kontant bruttolön, semesterersättning, provision | **X** | **X** | **X** | **X** | **X** | **X** | **X** | **X** | **X** | **X** | **X** | **X** | **X** |  |
| TLONT - Löneinkomst | **X** | **X** | **X** | **X** | **X** | **X** | **X** | **X** | **X** | **X** | **X** | **X** | **X** |  |
| TTJ - Inkomst av tjänst | **X** | **X** | **X** | **X** | **X** | **X** | **X** | **X** | **X** | **X** | **X** | **X** | **X** |  |
| NRV – Inkomst av näringsverksamhet | **X** | **X** | **X** | **X** | **X** | **X** | **X** | **X** | **X** | **X** | **X** | **X** | **X** |  |
| **In education** | | | | | | | | | | | | | |  |
| ISTUD- Studiemdel/studiehjälp, lån och bidrag | **X** | **X** | **X** | **X** | **X** | **X** | **X** | **X** | **X** | **X** | **X** | **X** | **X** |  |
| TUTBDOK – Utbildningsbidrag för doktorander | **X** | **X** | **X** | **X** | **X** | **X** | **X** | **X** | **X** | **X** | **X** | **X** | **X** |  |
| **Unemployed and/or on welfare benefit reliance** | | | | | | | | | | | | | |  |
| ISOCB - Ekonomiskt bistånd | **X** | **X** | **X** | **X** | **X** | **X** | **X** | **X** | **X** | **X** | **X** | **X** | **X** |  |
| TAGSTFA -  Ersättning som utgör inkomst av anställning | **X** | **X** | **X** | **X** |  | **X** | **X** | **X** | **X** | **X** | **X** | **X** | **X** |  |
| TFKASSA - Ersättning från försäkringskassa, inkomst av anställning | **X** | **X** | **X** | **X** | **X** | **X** | **X** | **X** | **X** | **X** | **X** | **X** | **X** |  |
| TKASSA - Dagpenning vid arbetslöshet | **X** | **X** | **X** | **X** | **X** | **X** | **X** | **X** | **X** | **X** | **X** | **X** | **X** |  |
| **Sickness (hospitalization and disability absence)** | | | | | | | | | | | | | |  |
| TREHAB -  Rehabilitetsersättning | **X** | **X** | **X** | **X** | **X** | **X** | **X** | **X** | **X** | **X** | **X** | **X** | **X** |  |
| TSA -  Sjuk och aktivitetsersättning | **X** | **X** | **X** | **X** | **X** | **X** | **X** | **X** | **X** | **X** | **X** | **X** | **X** |  |
| TSJUK -  Sjuk, havandeskaps, smittbärapenning samtsjuklönegaranti | **X** | **X** | **X** | **X** | **X** | **X** | **X** | **X** | **X** |  |  |  |  |  |
| TSJUKP (från 2013 ersätter TSJUK) -  Sjukpenning |  |  |  |  |  |  |  |  |  | **X** | **X** | **X** | **X** |  |

*For calculating employment income, the following income variables were subtracted from CSFVI: TUTBDOK, TSA, TREHAB, TKASSA, TFKASSA, TAGSTFA, PDEL, TPENSA, TPRIVL, TSJUK, TSJUKP.

Appendix Table 2. Cluster membership for women with and without PCOS diagnosis and for explanatory variables (%, reported within cluster), (**Sweden, 2025**).

|  | stable employment  T1(n=112,699) | education into employment  T2(n=15,599) | labor market exclusion  T3(n=12,109) | continuously unstable position  T4(n=8,794) | long-term sickness  T5(n=8,195) |
| --- | --- | --- | --- | --- | --- |
| **PCOS** | | | | | |
| No PCOS | 98.2% | 98.0% | 98.1% | 98.2% | 96.4% |
| Ever PCOS | 1.8% | 2.0% | 1.9% | 1.8% | 3.6% |
| **Civil status** | | | | | |
| Married | 27.1% | 33.4% | 28.2% | 35.2% | 24.2% |
| Not married | 72.9% | 66.6% | 71.8% | 64.8% | 75.8% |
| **Highest attained education** | | | | | |
| Primary | 4.2% | 2.9% | 15.7% | 14.7% | 18.9% |
| Secondary | 38.8% | 18.9% | 53.9% | 38.9% | 53.8% |
| Post-secondary | 57.0% | 78.3% | 30.4% | 46.4% | 27.3% |
| **Region of origin** | | | | | |
| Sweden | 87.0% | 73.1% | 74.1% | 61.6% | 85.2% |
| Outside of Sweden | 13.0% | 26.9% | 25.9% | 38.4% | 14.8% |
| **Children** | | | | | |
| No children | 54.0% | 41.7% | 36.2% | 59.2% | 48.7% |
| One or more children | 46.0% | 58.3% | 63.8% | 40.8% | 51.3% |

Appendix Figure 1. The quality of participation for different numbers of clusters (**Sweden, 2025**).

**
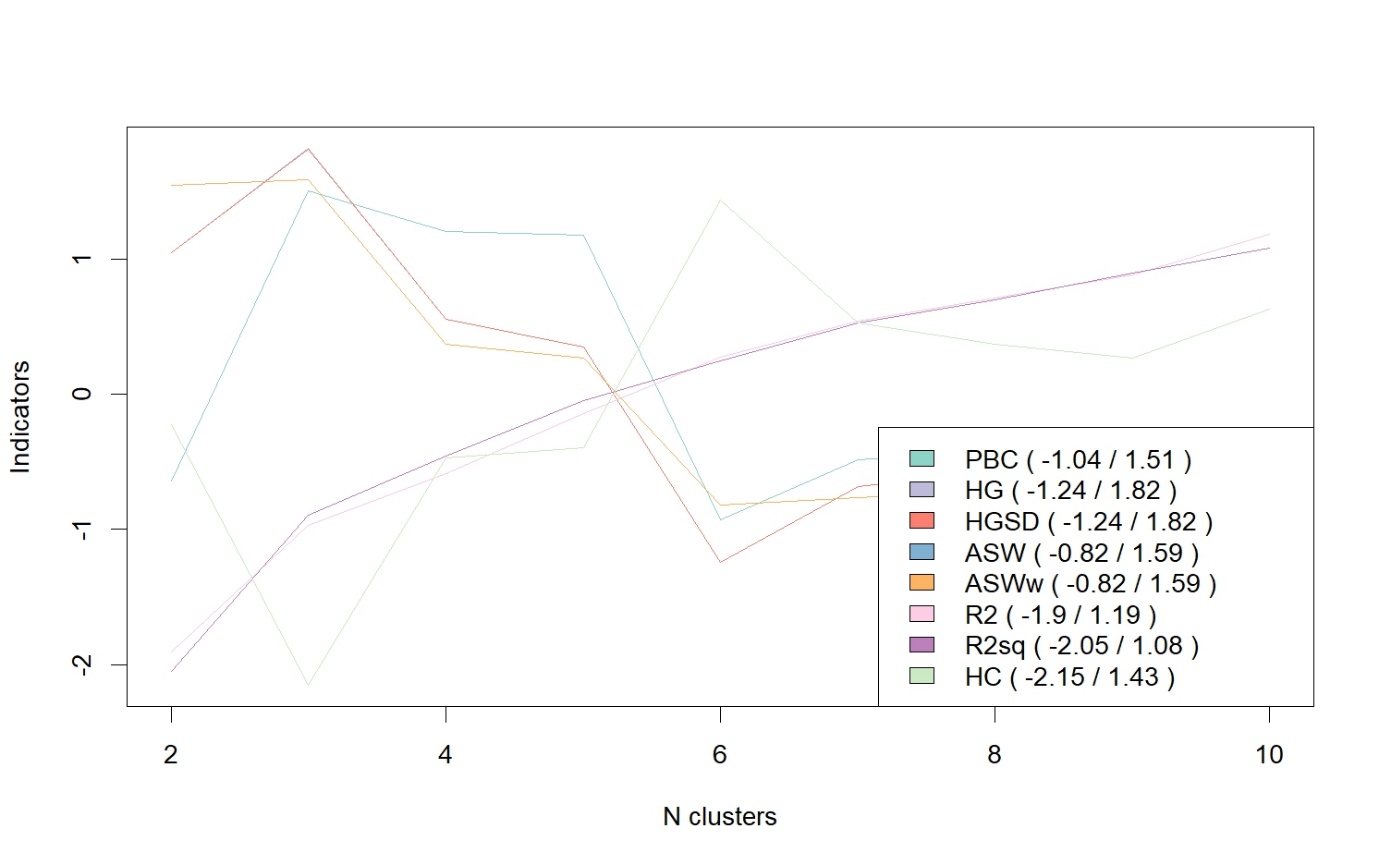
**
